# Point-of-care-ultrasound measurements of cardiovascular indices across awake, sedated, anesthetized, and recovered states in dogs

**DOI:** 10.3389/fvets.2026.1813463

**Published:** 2026-05-27

**Authors:** Jessie Warhoe, Kristin M. Zersen, Amanda A. Cavanagh

**Affiliations:** Department of Clinical Sciences, College of Veterinary Medicine and Biomedical Sciences, Colorado State University, Fort Collins, CO, United States

**Keywords:** anesthesia, caudal vena cava, dogs, hemodynamic monitoring, intravascular volume assessment, left ventricular end-diastolic diameter, point-of-care ultrasound

## Abstract

**Background:**

Point-of-care ultrasound (POCUS) assessment of intravascular volume in dogs commonly incorporates caudal vena cava (CVC) measurements, including the CVC-to-aorta ratio (CVC: Ao) and CVC collapsibility index (CVCCI). While these indices have been evaluated in awake dogs, the effects of sedation and anesthesia on these parameters remain unclear. Left ventricular end-diastolic diameter (LVEDD), a preload-sensitive echocardiographic measurement with established repeatability, may provide additional insight into cardiac loading conditions during anesthesia.

**Objective:**

To quantify CVC: Ao, CVCCI, and LVEDD in healthy dogs across four physiologic states and to evaluate the effects of anesthesia and systolic arterial pressure (SAP) on these indices, including intra-rater reliability.

**Animals:**

Eight purpose-bred, healthy adult neutered male Beagle dogs.

**Methods:**

In this prospective observational study, each dog underwent POCUS examination while awake, sedated, anesthetized, and recovered. CVC and aortic diameters were measured at paralumbar and iliac sites to calculate CVC: Ao; CVCCI was obtained from subxiphoid CVC measurements; and LVEDD was measured from a right parasternal short-axis view. SAP was recorded at each timepoint. Linear mixed-effects models assessed the effects of timepoint, SAP, and their interaction on ultrasound variables. Intra-rater reliability was evaluated using intraclass correlation coefficients.

**Results:**

SAP was lowest during anesthesia compared with awake and sedated states. LVEDD differed significantly by physiologic state and was smaller during anesthesia than during awake, sedated, or recovered states, while SAP was not an independent predictor of LVEDD. CVCCI varied across timepoints and demonstrated a strong positive association with SAP, with significant timepoint–SAP interactions. CVC: Ao ratios showed minimal or inconsistent changes across physiologic states. Intra-rater reliability was good for LVEDD, moderate to excellent for CVCCI, and poor to moderate for CVC: Ao measurements.

**Conclusions and clinical importance:**

In healthy dogs under anesthesia, LVEDD is a reproducible ultrasound measurement that reflects anesthesia-associated changes, independent of systolic arterial pressure, and should not be used independently to assess intravascular volume. CVCCI is strongly pressure-dependent under anesthesia and should not be interpreted as a standalone indicator of intravascular volume. CVC: Ao showed minimal state-dependent variation, however, it had lower repeatability, limiting its ability to assess intravascular volume in anesthetized dogs.

## Introduction

Point-of-care ultrasound (POCUS) has become an essential, noninvasive tool for evaluating intravascular volume and hemodynamic status in both human and veterinary critical care. Sonographic assessment of large vessels such as the caudal vena cava (CVC) and aorta (Ao) enables rapid bedside estimation of venous return and preload without the need for invasive monitoring (1, 2). In people, inferior vena cava (IVC) diameter and collapsibility index (IVCCI) are used to estimate right atrial pressure, guide fluid resuscitation, and predict post-induction hypotension (3). Comparable ultrasonographic indices in dogs—the caudal vena cava-to-aorta ratio (CVC: Ao) and CVC collapsibility index (CVCCI)—have shown promise as dynamic markers of volume status and fluid responsiveness (4–7). Accurate application of these indices, however, requires a clear understanding of their physiologic variability across anesthetic states and hemodynamic conditions.

Vena cava–derived indices are frequently used as noninvasive surrogates for intravascular volume assessment; however, this interpretation is inherently limited. These indices do not directly measure circulating blood volume, but instead reflect complex, dynamic interactions among venous return, transmural pressure, vascular tone, and intrathoracic pressure (1, 3). Their values are therefore highly dependent on physiologic context, including arterial blood pressure, respiratory mechanics, and patient state (e.g., awake versus anesthetized) (1–3). As a result, changes in these indices may not correspond directly to changes in intravascular volume and are more appropriately interpreted as integrative markers of cardiopulmonary physiology.

Several studies have characterized ultrasonographic CVC and CVC: Ao measurements in healthy, awake dogs, demonstrating feasibility and consistency across multiple imaging windows (8, 9). These indices decrease with experimentally induced hypovolemia and increase following fluid resuscitation, supporting their role as static indicators of intravascular volume (4–6, 10). In dogs with spontaneous circulatory shock or severe dehydration, CVC: Ao has been shown to increase following volume repletion, while marked elevations have also been reported in venous pressure–loaded states such as cardiac tamponade and chronic cardiac disease without overt right-sided heart failure (11, 12). Collectively, these findings indicate that CVC: Ao reflects changes in venous filling and transmural pressure but may be influenced by alterations in vascular compliance or changes in either vessel comprising the ratio. Accordingly, interpretation of CVC-derived indices requires careful consideration of underlying cardiovascular state and loading conditions, particularly across differing physiologic and anesthetic states.

Despite this recognized physiologic complexity, most veterinary studies evaluating CVC-derived indices have been performed in awake dogs or under limited and relatively controlled conditions (8, 9). Data evaluating these measurements under anesthesia remain limited. In mechanically ventilated dogs, the CVC: Ao ratio has been shown to correlate with systolic pressure variation but was not compared across anesthetic planes (13), while computed tomographic assessment has demonstrated only a weak association between CVC size and arterial pressure (14). In hypovolemic anesthetized dogs evaluated using a paralumbar CVC view, decreases in CVC diameter and CVC: Ao were observed with volume depletion (15), underscoring the diagnostic potential of these indices but also highlighting the need to account for anesthetic-related hemodynamic effects. Despite growing clinical use, the independent contributions of anesthesia and arterial pressure to sonographic venous measurements in dogs remain incompletely defined.

The physiologic effects of anesthetic drugs—including alterations in venous capacitance, sympathetic tone, and intrathoracic pressure—may further confound ultrasonographic estimates of central volume. CVCCI, a dynamic measure dependent on transmural pressure, is particularly susceptible to changes in arterial pressure and respiratory mechanics that may occur independently of true intravascular volume (3). Dynamic assessment of CVC diameter change during inspiration and expiration and calculation of caudal vena cava collapsibility index can predict fluid responsiveness, with greater percentage collapse during inspiration in those who are volume responsive. Because inhalant anesthesia induces vasodilation and myocardial depression, understanding whether CVC-derived indices behave predictably across anesthetic states is essential before applying them to guide fluid therapy in anesthetized patients.

Assessment of left ventricular (LV) dimensions provides complementary information regarding cardiac preload and loading conditions. Echocardiographic quantification of LV size and systolic function is well established in both dogs and people (16–20). Left ventricular end-diastolic diameter (LVEDD), measured from standardized echocardiographic views, is a preload-sensitive parameter and may decrease in response to anesthetic-induced reductions in venous return or myocardial relaxation. Consistent measurement technique is critical for detecting true physiologic change, as observer variability can meaningfully influence interpretation (21). Given these dependencies, establishing intra-rater repeatability for both CVC-based and LV measurements under controlled conditions is necessary to support their use in serial hemodynamic assessment.

The objective of the present study was to quantify ultrasonographic measurements of CVC: Ao, CVCCI, and LVEDD in healthy research dogs across awake, sedated, anesthetized, and recovery states, and to determine the relative effects of systolic arterial pressure and anesthesia on these indices. Intra-rater variability was evaluated using a single experienced operator to assess measurement repeatability across defined anatomic sites. We hypothesized that LVEDD would be affected by anesthesia but not arterial pressure, CVCCI would vary with arterial pressure, and CVC: Ao would be affected by both anesthesia and arterial pressure.

## Materials and methods

### Study design and animals

This was a prospective, observational study conducted using eight purpose-bred healthy adult Beagle dogs. The dogs (mean ± SD age 8.1 ± 0.8 years; mean ± SD weight 10.5 ± 1.5 kg) were all neutered males. Each dog underwent point-of-care ultrasound (POCUS) evaluations at four defined timepoints: (TP1) awake, (TP2) sedated, (TP3) anesthetized, and (TP4) recovered from anesthesia. This study was designed as a prospective physiologic investigation using a repeated-measures approach, in which each dog served as its own control across physiologic states. A formal sample size calculation was not performed. All procedures were approved by the Colorado State University Institutional Animal Care and Use Committee (IACUC; protocol number 6122).

### Inclusion and exclusion criteria

Dogs were included based on a normal physical examination, complete blood count, and serum biochemistry panel. Dogs were excluded if they demonstrated signs of clinical illness or hypovolemia. Dogs were prospectively designated for withdrawal from the study if they developed anesthesia-associated hypotension unresponsive to two 10 mL/kg boluses of lactated Ringer’s solution; no dogs met these criteria.

### Ultrasound protocol

Ultrasound examinations were performed using a Mindray M9Vet ultrasound system equipped with a 5-8 MHz microconvex probe. Dogs were gently restrained in right lateral recumbency for all measurements.

Caudal vena cava (CVC) and abdominal aortic (Ao) diameters were evaluated in the longitudinal plane using three standard transabdominal views: subxiphoid, paralumbar, and iliac. For assessment of the caudal vena cava collapsibility index (CVCCI), the maximum and minimum CVC diameters during inspiration and expiration were measured from the subxiphoid view and used to calculate CVCCI using the formula: [(CVC_max – CVC_min) / CVC_max] × 100. The CVC: Ao ratio was calculated using measurements acquired in the paralumbar (caudal to the right kidney) and iliac views (immediately rostral to the aortic bifurcation), where the vessels were imaged in parallel within the same scanning plane; measurements were obtained perpendicular to the vessel wall in a transverse orientation.

Left ventricular end-diastolic diameter (LVEDD), also referred to as left ventricular internal diameter in diastole (LVIDd), was measured using echocardiography from the right parasternal short axis view. Images were obtained perpendicular to the long axis of the left ventricle, at the level of the mitral valve leaflet tips, and measured at visually identified end-diastole based on maximal ventricular dimension. The internal dimension of the left ventricle was recorded across the minor axis at the chordal level.

For all ultrasound measurements, each parameter was obtained by repeating the full process of image acquisition, frame capture, and measurement three times in real time during the examination. This repeated measures approach was used to assess intra-rater repeatability and to reduce physiologic variability related to beat-to-beat and respiratory effects. Measurements were not synchronized with electrocardiography; therefore, repeated acquisition and averaging were used to minimize variability associated with cardiac cycle phase.

**Figure 1:**
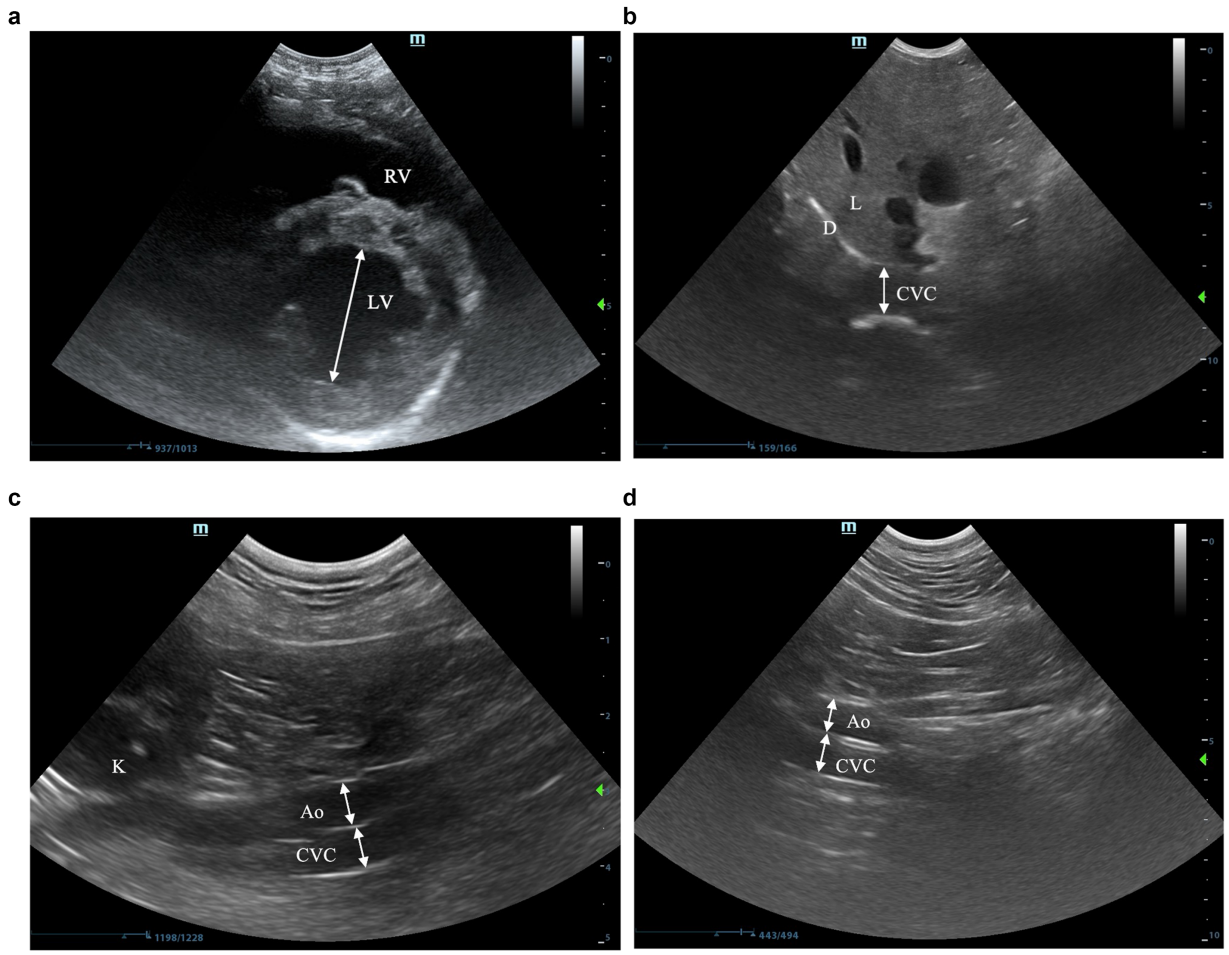
Representative ultrasound images demonstrating measurement techniques for point-of-care ultrasonographic indices. **(a)** Right parasternal short-axis view of the left ventricle (LV) used for measurement of left ventricular end-diastolic diameter (LVEDD), with right ventricle (RV) visible. **(b)** Subxiphoid view of the caudal vena cava (CVC) used for calculation of the caudal vena cava collapsibility index (CVCCI), with liver (L) and diaphragm (D) identified. **(c)** Paralumbar view of the caudal vena cava and aorta (Ao) used for calculation of the CVC: Ao ratio, with kidney (K) identified. **(d)** Iliac view of the caudal vena cava and aorta used for calculation of the CVC: Ao ratio.

For all measurements, electronic calipers were used on the ultrasound console with an inner-to-outer edge technique. One trained and experienced investigator performed the ultrasound measurements for each dog at each timepoint, while a second investigator assisted with documentation during the examination. Representative ultrasound views and measurement locations for LVEDD, CVCCI, and CVC: Ao measurements are shown in Figure 1.

### Experimental protocol

Baseline data including body weight, age, and sex were recorded. Heart rate (HR), respiratory rate (RR), rectal temperature, and systolic arterial pressure (SAP) were recorded at each timepoint. SAP was obtained using Doppler ultrasonography for all non-anesthetized timepoints, consistent with standard clinical practice in conscious dogs. TP1 ultrasound measurements were then performed as described.

Dogs were sedated with butorphanol (0.3 mg/kg intramuscularly), selected as a commonly used sedative in dogs with minimal expected effects on cardiovascular function at clinically relevant doses, and allowed to equilibrate for 10 min prior to TP2 measurements to permit stabilization of physiologic parameters. Dogs were subsequently preoxygenated with 100% FiO₂ via face mask, and anesthesia was induced with propofol (5–10 mg/kg intravenously to effect), followed by orotracheal intubation and maintenance with isoflurane in 100% FiO₂ delivered via a circle rebreathing system. This anesthetic protocol was selected to reflect standard clinical practice and to allow controlled induction and maintenance of anesthesia.

Dogs remained spontaneously breathing throughout anesthesia. Standard continuous monitoring was employed, including electrocardiogram, pulse oximetry, end-tidal CO₂, temperature monitoring, and invasive arterial blood pressure monitoring via arterial catheterization. Anesthetic depth was titrated to maintain a consistent, clinically appropriate plane based on standard monitoring parameters, but was not formally quantified using a standardized scoring system. During anesthesia, systolic, diastolic, and mean arterial pressures were recorded at 10-min intervals; however, only SAP was used for analyses to maintain consistency across timepoints. Invasive arterial monitoring was used during anesthesia to allow continuous and direct measurement of arterial pressure under conditions in which Doppler measurements may be less reliable. After a 10-min equilibration period under general anesthesia, TP3 ultrasound measurements were obtained to allow stabilization of anesthetic and hemodynamic effects.

Dogs were monitored during recovery and TP4 measurements were obtained 30 min after dogs became ambulatory and vital signs had normalized.

### Statistics

Intra-rater variability was assessed for each ultrasonographic variable using repeated measurements obtained by a single experienced operator across the four study timepoints. Intraclass correlation coefficients (ICC) were calculated to quantify within-operator measurement repeatability for each variable at each timepoint.

Linear mixed-effects models were used to compare ultrasonographic parameters across timepoints and measurement locations, including interaction terms where appropriate. Systolic arterial pressure was not analyzed as a primary outcome variable across physiologic states but was included as a covariate in all models to evaluate its influence on sonographic measurements and potential interactions with timepoint. Dog identification number was included as a random effect to account for repeated measurements within subjects. Statistical significance was defined as *p* < 0.05. All statistical analyses were performed using SAS version 9.4 (SAS Institute Inc., Cary, NC). ICC calculations were performed using MedCalc version 23.1.6 (MedCalc Software Ltd., Ostend, Belgium).

## Results

**Figure 2:**
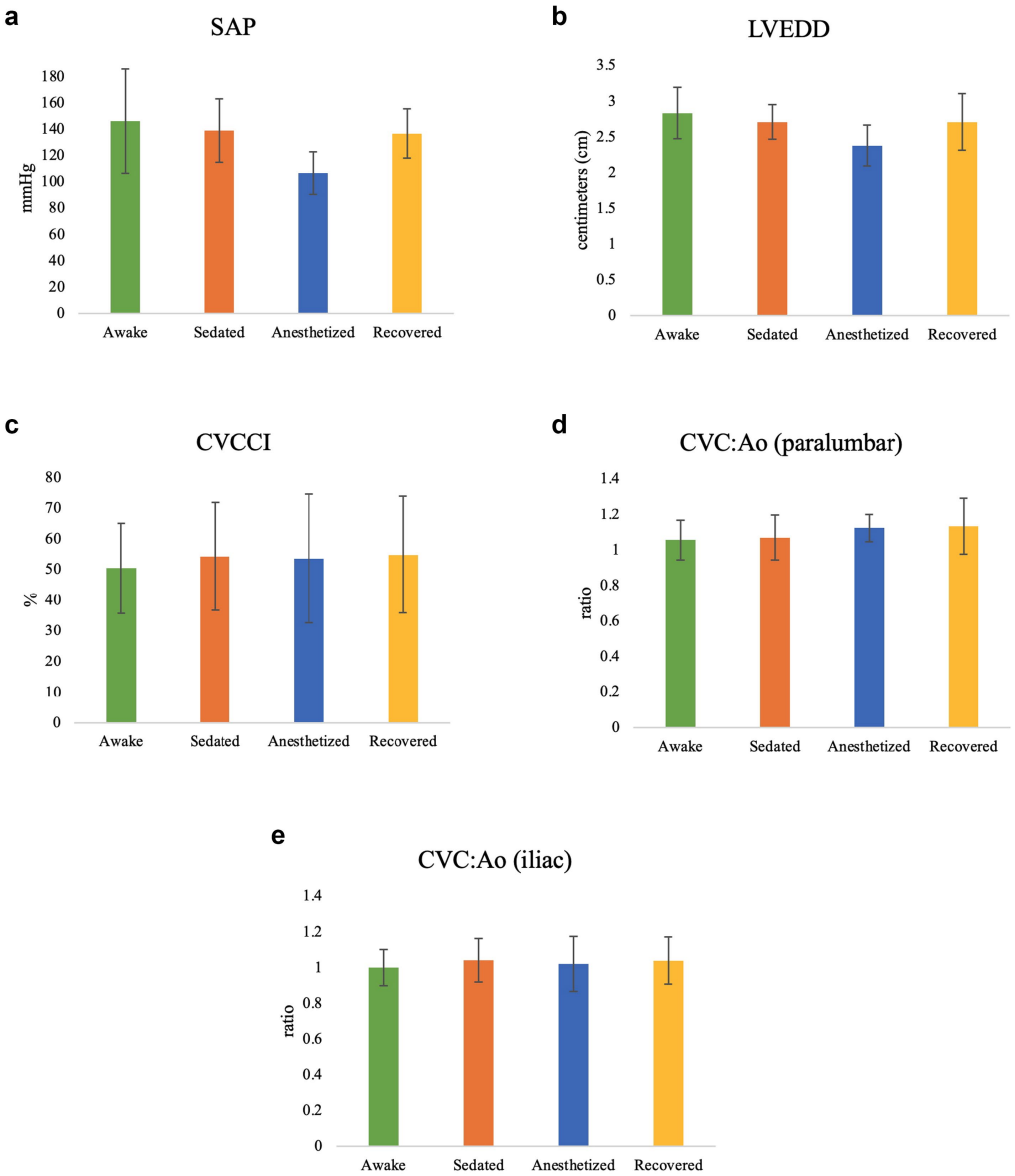
Timepoint-dependent changes in sonographic indices across physiologic states. Bar charts depict mean ± standard deviation for **(a)** Systolic arterial pressure (SAP, mmHg), **(b)** Left ventricular end-diastolic diameter (LVEDD, cm), **(c)** Caudal vena cava collapsibility index (CVCCI, %), **(d)** Caudal vena cava to aorta ratio measured at the paralumbar site (CVC: Ao, paralumbar), and **(e)** Caudal vena cava to aorta ratio measured at the iliac site (CVC: Ao, iliac) in dogs evaluated while awake, sedated, anesthetized, and recovered.

**Figure 3:**
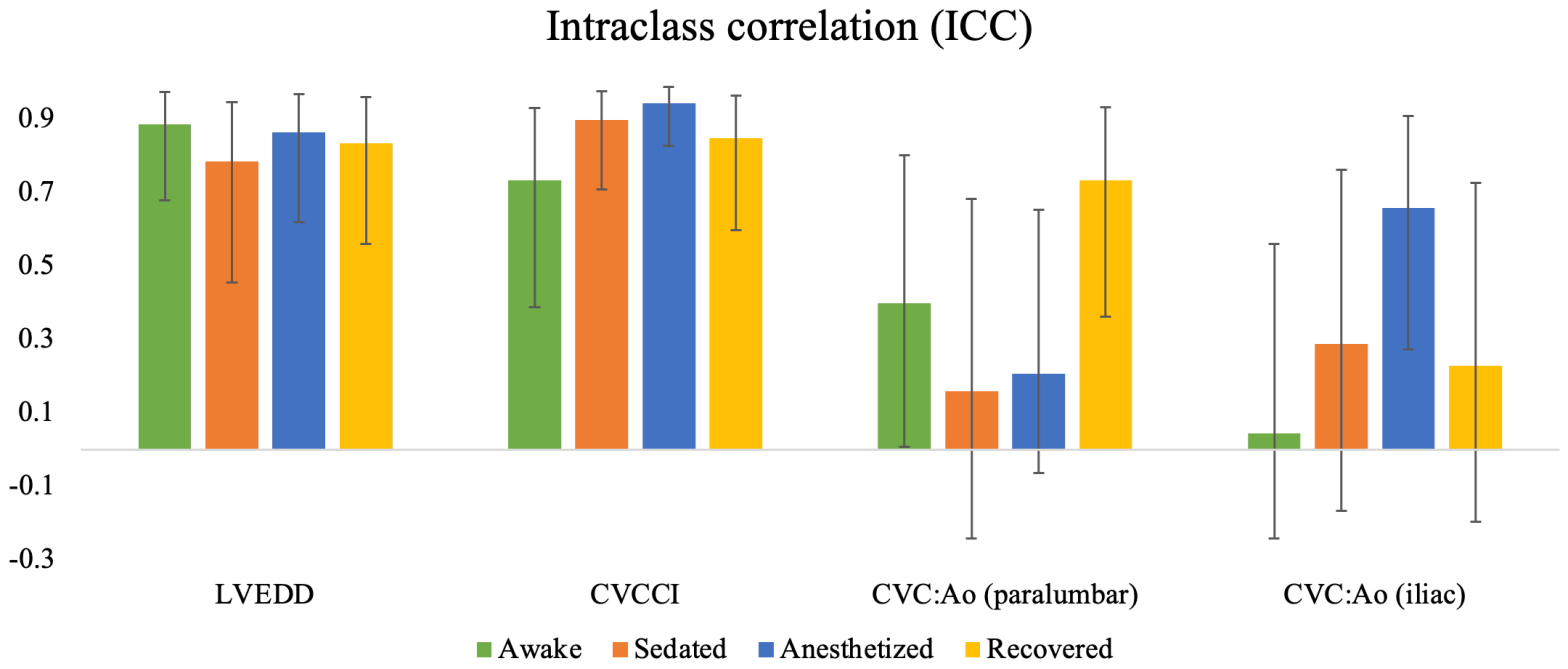
Intra-rater reliability of sonographic measurements across physiologic states. Bar charts depict intraclass correlation coefficients (ICC) with 95% confidence intervals for LVEDD, CVCCI, CVC: Ao (paralumbar), and CVC: Ao (iliac) in dogs evaluated while awake, sedated, anesthetized, and recovered.

Descriptive statistics, model-based *p*-values, and intra-rater reliability estimates for all ultrasonographic measurements across physiologic states are summarized in Table 1, with mean ± SD values illustrated in Figure 2 and intra-rater reliability (ICC) results shown in Figure 3.

**Table 1:** Ultrasonographic measurements (mean ± SD) and intra-rater reliability across physiologic states.

Parameter	Unit	Awake	Sedated	Anesthetized	Recovered	Type III p (time)	ICC (95% CI)	Reliability
SAP	mmHg	145.8 ± 39.6	138.8 ± 24.0	106.3 ± 16.1	136.4 ± 18.8	—	—	—
LVEDD	cm	2.828 ± 0.359	2.704 ± 0.243	2.375 ± 0.286	2.705 ± 0.398	<0.0001	Aw: 0.886 (0.679–0.974) S: 0.785 (0.455–0.947) An: 0.865 (0.620–0.969) R: 0.834 (0.561–0.960)	Good (all)
CVCCI	%	50.30 ± 14.67	54.20 ± 17.59	53.53 ± 20.88	54.74 ± 18.98	<0.0001	Aw: 0.734 (0.387–0.932) S: 0.898 (0.709–0.977) An: 0.943 (0.828–0.987) R: 0.848 (0.599–0.964)	Excellent/Good–Moderate
CVC: Ao (paralumbar)	Ratio	1.053 ± 0.112	1.067 ± 0.127	1.121 ± 0.077	1.132 ± 0.159	0.878 (NS)	Aw: 0.399 (0.008–0.801) S: 0.159 (−0.242–0.683) An: 0.207 (−0.065–0.654) R: 0.734 (0.327–0.933)	Moderate (Recovered), Poor (others)
CVC: Ao (iliac)	Ratio	0.999 ± 0.101	1.041 ± 0.122	1.018 ± 0.154	1.038 ± 0.132	0.0318	Aw: 0.043 (−0.243–0.561) S: 0.288 (−0.167–0.762) An: 0.658 (0.273–0.908) R: 0.228 (−0.197–0.725)	Moderate (Anesth), Poor (others)

Aw, Awake; S, Sedated; An, Anesthetized; R, Recovered; ICC reported per timepoint for the single operator. Type III *p*-values are from mixed models. Reliability interpretation based on established intraclass correlation coefficient thresholds (23).

### Hemodynamics

SAP values varied across physiologic states, with lower values during anesthesia (106.3 ± 16.1 mm Hg; n = 8) compared with awake (145.8 ± 39.6 mm Hg; n = 8), sedated (138.8 ± 24.0 mm Hg; n = 8), and recovery post-anesthesia (136.4 ± 18.8 mm Hg; n = 8).

### LVEDD

Mean ± SD LVEDD values were 2.828 ± 0.359 cm (awake), 2.704 ± 0.243 cm (sedated), 2.375 ± 0.286 cm (anesthetized), and 2.705 ± 0.398 cm (recovered). LVEDD differed significantly across timepoints (Type III *p* < 0.0001). In the timepoint-only model, LVEDD was lower during anesthesia compared with awake, sedated, and recovered states; other comparisons were not significant.

When SAP was included in the model, the main effect of SAP on LVEDD was not significant (estimate 0.0019 cm per mm Hg, *p* = 0.287), while the SAP × timepoint interaction remained significant (Type III *p* < 0.0001). Pairwise contrasts adjusted for SAP confirmed lower LVEDD during anesthesia compared with awake, sedated, and recovered states. Intra-rater reliability for LVEDD was good at all timepoints (ICC 0.785–0.886).

### CVCCI

Mean ± SD CVCCI values were 50.30 ± 14.67% (awake), 54.20 ± 17.59% (sedated), 53.53 ± 20.88% (anesthetized), and 54.74 ± 18.98% (recovered). CVCCI differed by physiologic state in the timepoint-only model (Type III *p* < 0.0001), with higher values during anesthesia compared with awake and sedated states, and lower values during awake compared with recovery.

In models including SAP, the main effect of SAP on CVCCI was significant (estimate +0.452% per mm Hg, p < 0.0001), and a significant SAP × timepoint interaction was present (Type III *p* = 0.0108). Intra-rater reliability for CVCCI ranged from moderate to excellent across timepoints (ICC 0.734–0.943).

### CVC: Ao (paralumbar view)

Mean ± SD paralumbar CVC: Ao ratios were 1.053 ± 0.112 (awake), 1.067 ± 0.127 (sedated), 1.121 ± 0.077 (anesthetized), and 1.132 ± 0.159 (recovered). The timepoint effect was not significant in models without SAP (Type III *p* = 0.878), and neither the SAP main effect nor the SAP × timepoint interaction was significant in adjusted models. Intra-rater reliability was poor at most timepoints and moderate during recovery.

### CVC: Ao (iliac view)

Mean ± SD iliac CVC: Ao ratios were 0.999 ± 0.101 (awake), 1.041 ± 0.122 (sedated), 1.018 ± 0.154 (anesthetized), and 1.038 ± 0.132 (recovered). The timepoint effect was significant in the timepoint-only model (Type III *p* = 0.0318), driven by a difference between awake and sedated states. Timepoint remained significant after adjustment for SAP, with significant SAP × timepoint interactions involving the recovered state. Intra-rater reliability was moderate under anesthesia and poor at other timepoints.

## Discussion

This prospective study evaluated the effects of sedation, general anesthesia, and recovery on commonly used point-of-care ultrasound (POCUS) indices of intravascular volume and cardiac loading in healthy dogs. The principal findings were that left ventricular end-diastolic diameter (LVEDD) decreased consistently under general anesthesia independent of blood pressure; caudal vena cava collapsibility index (CVCCI) was strongly associated with blood pressure and demonstrated significant state-dependent interactions; caudal vena cava–to–aorta (CVC: Ao) ratios showed minimal or inconsistent changes across physiologic states with generally low intra-rater reliability. Collectively, these results indicate that while some POCUS-derived indices reliably reflect anesthesia-associated cardiovascular changes, others are meaningfully influenced by pressure dynamics or measurement variability under anesthesia. Rather than serving as direct measures of intravascular volume, these ultrasonographic indices reflect dynamic interactions among venous filling, vascular tone, cardiac compliance, and anesthetic state. Their clinical utility therefore lies in informed, context-specific interpretation integrated with concurrent hemodynamic findings, rather than reliance on isolated numerical thresholds.

While echocardiographic assessment of individual cardiovascular parameters is well established, the novelty of this study lies in the controlled comparison of multiple POCUS-derived indices that are commonly interpreted together in clinical practice, including LVEDD and CVC-derived measurements, across physiologic states within the same cohort, while simultaneously accounting for the influence of blood pressure and measurement repeatability. This approach provides clinically relevant insight into the relative interpretability of these indices when used in combination under conditions commonly encountered in veterinary critical care.

LVEDD decreased significantly during anesthesia compared with awake, sedated, and recovered states, even after accounting for variation in blood pressure. This finding aligns with well-established physiologic effects of inhalant anesthetics, including reductions in venous return, decreased sympathetic tone, and alterations in ventricular relaxation and compliance, all of which contribute to reduced cardiac preload and smaller diastolic chamber dimensions. Prior echocardiographic studies in dogs and people have demonstrated the sensitivity of LVEDD to changes in loading conditions and myocardial properties (16–20). Importantly, the absence of an independent association between blood pressure and LVEDD in the present study suggests that the observed reductions in LVEDD under anesthesia reflect global changes in cardiac loading rather than short-term pressure fluctuations.

These findings have important implications for clinical interpretation. Although LVEDD is reproducible and responds predictably to anesthetic state, changes in LVEDD obtained under anesthesia should not be interpreted as direct indicators of intravascular volume or compared directly with measurements obtained in awake dogs. Rather, LVEDD appears to function best as a stable, internally consistent physiologic reference for assessing relative changes in cardiac loading within a given physiologic state, reflecting composite effects of preload, ventricular compliance, and anesthetic modulation rather than volume status alone. The good intra-rater reliability observed across all timepoints further supports its utility for serial assessment during anesthetic procedures, provided measurements are interpreted within appropriate physiologic context. When interpreted alongside concurrently obtained CVC-derived indices, these findings support the use of LVEDD as a stable reference component within a multi-parameter POCUS assessment, rather than as a standalone indicator of intravascular volume.

In contrast, CVCCI demonstrated marked dependence on blood pressure and significant timepoint–pressure interactions. This finding is physiologically intuitive, as collapsibility indices reflect the dynamic response of the compliant venous system to changes in transmural pressure, which are influenced by right atrial pressure, intrathoracic pressure, and vascular tone. Under anesthesia, reductions in sympathetic tone and venous capacitance, combined with altered respiratory mechanics, can exaggerate respiratory variation in CVC diameter even in the absence of true hypovolemia. Although intra-rater reliability for CVCCI was good to excellent, the strong pressure dependence observed in this study indicates that CVCCI primarily reflects venous pressure dynamics rather than intravascular volume alone under anesthesia.

These results are consistent with prior veterinary and human literature demonstrating that collapsibility indices are most informative in spontaneously breathing, normotensive patients and become unreliable when vascular tone or intrathoracic pressures are altered (1, 3, 4, 6, 7). The present findings reinforce this limitation in anesthetized dogs, suggesting that CVCCI should be interpreted alongside concurrent hemodynamic parameters rather than used in isolation to infer volume status during anesthesia, particularly in hypotensive or low-pressure states. Dynamic assessment of preload and prediction of fluid responsiveness may provide additional clinically relevant information; however, these parameters were not evaluated in the present study.

These findings also highlight the important distinction between assessment of intravascular volume and prediction of fluid responsiveness. While CVC-derived indices and LVEDD are often used as surrogate markers of volume status, they do not directly predict whether a patient will respond to fluid administration. Fluid responsiveness depends on dynamic cardiovascular interactions and cannot be inferred from static or single-point measurements alone. Accordingly, the indices evaluated in this study should not be used in isolation for clinical decision-making, but rather interpreted in conjunction with the overall clinical picture, including perfusion parameters, blood pressure, and trends over time. Reliance on isolated measurements may be particularly misleading under anesthesia, where vascular tone and cardiopulmonary interactions are altered.

CVC: Ao ratios demonstrated modest and inconsistent changes across physiologic states, with no significant anesthesia-related differences at the paralumbar site and only limited timepoint effects at the iliac site. Adjustment for blood pressure did not reveal consistent pressure dependence at either measurement location. Intra-rater reliability for CVC: Ao measurements was poor-to-moderate across physiologic states, particularly at the paralumbar site. This variability likely reflects sensitivity of ratio-based indices to probe orientation, vessel boundary definition, respiratory phase, and small absolute measurement differences, all of which can disproportionately influence derived ratios (2, 21). These findings suggest that although CVC: Ao ratios did not vary across physiologic states and therefore may be a better marker of intravascular volume, it’s poor intra-rater reliability limits its ability to assess intravascular volume in anesthetized dogs.

This comparatively modest variation contrasts with prior studies performed in awake or clinically unstable dogs, in which CVC: Ao ratios decreased during hypovolemia and increased following fluid resuscitation in conditions such as parvoviral enteritis, spontaneous circulatory shock, and cardiac tamponade (5, 6, 10, 12). Additionally, increased CVC diameters and CVC: Ao ratios have been reported in dogs with chronic volume loading or elevated venous pressures, including degenerative mitral valve disease without overt right-sided heart failure (11). Together, these studies suggest that CVC: Ao is responsive to large or pathologic shifts in venous filling and transmural pressure.

Several limitations warrant consideration. The study population consisted exclusively of healthy, purpose-bred male Beagle dogs, which may limit generalizability to other breeds, ages, or dogs with cardiovascular or systemic disease. A formal sample size calculation was not performed, which may limit assessment of statistical power; however, the repeated-measures study design allowed each subject to serve as its own control, improving detection of within-subject changes across physiologic states. Although dogs were screened based on physical examination and routine laboratory testing, comprehensive baseline echocardiography was not performed, and subclinical cardiac abnormalities cannot be definitively excluded. Ultrasound measurements were not synchronized with electrocardiography, and identification of end-diastole was based on visual assessment of maximal ventricular dimension, which may introduce variability related to cardiac cycle phase, particularly in dogs with respiratory sinus arrhythmia; however, repeated measurements and averaging were used to mitigate this effect. Anesthetic depth was not formally standardized using a defined scoring system, and although a consistent clinical plane of anesthesia was maintained, variation in anesthetic depth may have influenced cardiovascular parameters. Systolic arterial pressure was measured using different techniques across physiologic states (Doppler ultrasonography in non-anesthetized dogs and invasive arterial monitoring during anesthesia). Although these approaches reflect standard clinical practice, systematic differences between techniques cannot be excluded and may have influenced observed associations with ultrasound-derived variables. The study design focused on within-operator measurement variability to isolate physiologic effects from operator-dependent sources of variation; inter-rater agreement remains an important area for future investigation, particularly given reported variability between operators for select CVC-derived measurements even after focused training and for broader clinical application of POCUS protocols (22). Invasive measures of preload or cardiac output were not obtained, limiting direct correlation between ultrasound-derived indices and gold-standard hemodynamic parameters. The recovery timepoint may also have been influenced by residual anesthetic or sedative effects.

Future studies should evaluate these indices in clinically diverse canine populations, including hypovolemic, septic, and patients with cardiac disease, and assess how disease states interact with anesthetic-induced changes in cardiovascular physiology. Incorporation of inter-rater reliability analyses, standardized acquisition protocols, and comparisons with invasive or advanced echocardiographic measures of preload and cardiac output will be essential to refine clinical interpretation.

In conclusion, these findings underscore the importance of context-specific interpretation of POCUS-derived hemodynamic indices during anesthesia. LVEDD emerged as a reproducible measurement in anesthetized dogs that changed systematically with anesthetic state while remaining independent of blood pressure. Accordingly, LVEDD measured under anesthesia should not be interpreted as a direct indicator of intravascular volume, used in isolation to infer volume status, or compared directly with measurements obtained in awake dogs. CVCCI was strongly influenced by blood pressure under anesthesia, indicating that collapsibility primarily reflects venous pressure dynamics, and should not be used independently to assess intravascular volume. CVC: Ao ratios showed limited changes in anesthetic states, suggesting it may be a good marker of intravascular volume, however, poor repeatability makes this measurement less reliable. Together, these findings emphasize that POCUS-derived hemodynamic indices reflect integrated cardiovascular physiology, and their clinical utility is maximized when interpreted collectively within a multi-parameter, context-specific assessment framework rather than as isolated measurements.

## Data Availability

The raw data supporting the conclusions of this article will be made available by the authors, without undue reservation.
